# Evaluation of cognitive impairment in a French sample of patients with restrictive anorexia nervosa: two distinct profiles emerged with differences in impaired functions and psychopathological symptoms

**DOI:** 10.1007/s40519-020-00981-w

**Published:** 2020-08-07

**Authors:** J. Cholet, M. Rousselet, Y. Donnio, M. Burlot, M. Pere, S. Lambert, B. Rocher, M. Chirio-Espitalier, E. Eyzop, M. Grall-Bronnec

**Affiliations:** 1 Clinical Investigation Unit BALANCED “BehaviorAL AddictioNs and ComplEx Mood Disorders”, Addictology and Psychiatry Department, University Hospital of Nantes, 85 Rue Saint Jacques, 44093 Nantes Cedex 1, France; 2 U1246 SPHERE “methodS in Patient-Centered Outcomes and HEalth ResEarch”, INSERM, University of Nantes and Tours, 22 Boulevard Benoni-Goullin, 44200 Nantes, France; 3 Biostatistics Unit, Research Board, Nantes University Hospital, 5 Allées de l’île Gloriette, 44093 Nantes Cedex 1, France; 4 Reference Centre for Therapeutic Education and Cognitive Remediation Care (CReSERC), Psychiatry and Mental Health Department, University Hospital of Nantes, 85 Rue Saint Jacques, 44093 Nantes Cedex 1, France

**Keywords:** Anorexia nervosa, Cognition, Executive functions, Cognitive flexibility, Intelligence quotient

## Abstract

**Purpose:**

The cognitive profiles of patients suffering from anorexia nervosa (AN) are currently explored as potential facilitating and/or maintenance factors. Specific data in restrictive AN (AN-R) remain contradictory. This study focused on women with AN-R to evaluate their cognitive functions to develop a more specific cognitive remediation program.

**Methods:**

Female patients older than 15 years who were suffering from AN-R were recruited in a specialized unit for eating disorder management. Female healthy control (HC) participants were recruited who were matched with AN patients on age. All participants completed a cognitive evaluation (premorbid intelligence quotient (IQ), planning, information processing speed, cognitive flexibility) and a clinical evaluation (impulsivity, anxiety, depression).

**Results:**

A total of 122 participants were included. Patients suffering from AN-R had significant impairment in information processing speed and planning. Patients exhibited significantly better cognitive flexibility than did the HC group when adjustments were made for other cognitive functions and impulsivity. Two distinct subgroups of patients were identified. The first subgroup had more marked cognitive impairment and fewer psychopathological symptoms than did the second subgroup of patients and the HC group.

**Conclusion:**

Our results highlight cognitive impairment in patients with AN who had normal premorbid IQ. Two distinct profiles emerge. In clinical practice, these results open up perspectives for the development of more specific cognitive remediation programs (one specific program for cold cognitions and another specific program targeting emotions and hot cognitions). These results warrant confirmation by larger studies with a more specific evaluation of the impact of emotional status.

**Trial registration** NTC02381639, Date of registration. March 6, 2015

**Electronic supplementary material:**

The online version of this article (10.1007/s40519-020-00981-w) contains supplementary material, which is available to authorized users.

## Introduction

As a serious pathology of adolescence, anorexia nervosa (AN) represents an important public health issue. The current model of AN is multifactorial, involving predisposing, precipitating and maintenance factors of the disorder with individual factors (genetic and psychological) in close interaction with socioenvironmental factors (family, environmental and sociocultural factors). For this reason, several studies have examined the cognitive profiles of patients as a potential facilitating factor (with the question of a cognitive endophenotype) but also as a maintenance factor of this eating disorder [1, 2]. Specific data in restrictive AN (AN-R) remain contradictory, mainly because of the clinical heterogeneity of the included patients. Unfortunately, few studies specifically evaluate only patients with AN-R, despite the consensus recommendations to distinguish patients with AN-R from those with AN with purging and/or bulimic types [3, 4]. Recent literature data, focused on patients suffering from AN, would converge towards an impairment in executive function (i.e., mental flexibility [5] cognitive inhibition [6] and decision-making [7]). More recently the research had focused on central coherence [8], which is a complex cognitive capacity for the integration of information into a global meaning. The results tend to converge towards attainment of central coherence, which some authors link with clinical specificities, such as the morbid pursuit of thinness and body image distortions, or such as traits of autistic spectrum disorders or obsessive traits [9]. Additionally, recent data support the attainment of visuospatial abilities, regardless of a perceptual disorder [10]. More recently, the impact of emotional status (depression in particular) has complemented the current models and research perspectives [11].

The question of the cognitive impact of undernutrition is still debated. However, the data seem to converge towards a consensus: if undernutrition has cognitive consequences (especially on attentional abilities), the impairment of executive functions seems to persist in patients without clinical symptoms of AN-R but who are still underweight (i.e., with a body mass index lower than 21 according to the standards of the World Health Organization of 2008) [12, 13].

Thus, recent data highlight difficulties in adapting to external stimuli (mental flexibility), preventing automatic responses (cognitive inhibition) and integrating different information (decision-making), regardless of the stage of disease (active or resolute phase). For some researchers, the disorders found were mainly due to a state of hypervigilance that would saturate the working memory of information and prevent patients from filtering information and adapting to environmental stimuli [14].

The aim of the study was to—(a) replicate previous data regarding extensive cognitive domains (in particular dysexecutive functions and central coherence) in a sample of French patients with AN-R and (b) examine whether differences in neuropsychological test performance are associated with clinical characteristics (psychiatric comorbidities, impulsivity, addictive comorbidities). We hypothesized that cognitive impairment would be confirmed in patients with AN-R and that differences in cognitive profiles would be independent of clinical characteristics.

## Methods

### Procedure and ethics

Patients with anorexia nervosa (AN) were recruited in a specialized unit for eating disorder (ED) management that provides physical, psychological and social care in accordance with the guidelines for ED management [15, 16]. Treatment is primarily conducted in an outpatient format, with inpatient treatment provided only if necessary. Healthy control (HC) participants were recruited via media announcements (newspapers, radio and websites); these HC participants were matched with AN patients for age according to groupwise criteria based on norms for cognitive evaluation [17, 18]. A preselection interview was conducted by phone by the research staff to assess the inclusion and exclusion criteria. The research staff also verified that the HC and AN groups were age-matched and scheduled appointments for the evaluation. For each participant (HC and AN), self-questionnaires were sent or handed (for AN patients who were hospitalized), and the participant had to complete the questionnaires before the evaluation so that the research staff could verify that they were properly completed at the beginning of the evaluation. The evaluation was the same for the HC and AN participants; it took place in a face-to-face setting with a staff research member. The evaluation lasted 1 h and a half. For hospitalized patients (i.e., physically weaker), the interview was scheduled immediately after rest times. If the patient was not able to perform the evaluation according to the medical staff, the evaluation was deferred to another day. The evaluation started with the verification of ED diagnosis (no ED diagnosis for HCs and AN-R for AN patients) and the cognition portion to ensure that the participant was in the best condition possible. Then, psychiatric comorbidities were assessed, and anthropometric measures were performed. All diagnoses (ED and psychiatric comorbidities) were made according to DSM IV criteria in a face-to-face interview using the Mini International Neuropsychiatric Interview (MINI). The tools and measures are detailed in a dedicated paragraph. Participants were informed that they could stop the evaluation at any moment if they felt exhausted. Only one participant (AN group) asked for a break. The second portion (psychiatric comorbidities) was performed 1 week later.

This study was approved by the French Research Ethics Committee (CPP) on April 2, 2015. All participants (AN group and HC group) provided written informed consent (for those under 18 years old, written informed consent was provided by a legal representative) in accordance with the Declaration of Helsinki. The study is registered as NCT02381639.

### Participants

#### AN group

All participants were women meeting the DSM-IV criteria for a current diagnosis of AN restricting type [19], over 15 years old and treated in a specialized unit for ED. Patients with current nasogastric renutrition, a personal history of diseases that could affect cognitive evaluation (head trauma, neurodegenerative diseases, uncontrolled epilepsy, uncontrolled endocrine disorder and mental retardation) or past or current treatment with cognitive remediation were not included. Recruitment was carried out between June 2015 and July 2017.

#### HC group

All participants were women over 15 years old without a history of ED or current ED according to DSM-IV criteria and the Eating Attitude Test-26 (EAT-26) (EAT total score < 20). Similar to the AN group, participants with a personal history of diseases that could affect cognitive evaluation and past or current treatment with cognitive remediation were not included. The recruitment of HC participants took place between February 2016 and August 2017.

A total of 122 participants were included (62 AN and 60 HC). Three participants were excluded from the AN group: two patients consented to participate in the study but ultimately refused to participate when we called them to schedule the evaluation, and one patient was currently engaged in purging behavior. One participant from the HC group was excluded because she had a history of bulimia nervosa that was not detected during the preselection interview. Finally, 59 patients were included in the AN group, and 59 age-matched participants were included in the HC group. The mean age of the total sample was 21.0 years (SD 5.7), 20.7 years (SD 5.4) for AN group and 21.3 years (SD 6.0) for the HC group.

### Measures

#### Anthropometric measure

Body mass index (BMI). On the day of the cognitive evaluation, height and weight were used to measure current BMI; the same scale was used for all participants.

#### Psychiatric morbidities

Mini-International Neuropsychiatric Interview (MINI). The fifth version of the MINI is a structured diagnostic interview that enables rapid and systematic investigations of the main axis 1 psychiatric disorders [20]. The kappa coefficient, sensitivity and specificity were good or very good for all diagnoses with the exception of a generalized anxiety disorder (GAD) (kappa = 0.36), agoraphobia (sensitivity = 0.59) and bulimia (kappa = 0.53). For the purpose of the study, a French version was used [21]. We assessed current mood disorders (major depressive episode, dysthymia, (hypo-)manic episodes), current suicidal risk, current anxiety disorders (panic disorder, agoraphobia, social phobia, obsessive-compulsive disorder, posttraumatic stress disorder, generalized anxiety disorder), current substance use disorders (alcohol, tobacco, illicit substances and medicines) and EDs. The ED sections were administered to both the AN group and the HC group before participants were included in the study to ensure that the AN restriction type was the current ED diagnosis for patients from the AN group and to ensure that participants from the HC group did not have any history of ED or current ED.

#### Eating disorders characteristics

The Eating Attitude Test-26 (EAT-26) is a 26-item self-questionnaire [22] validated in French [23]. This self-assessment questionnaire assesses a broad range of symptoms and provides a total score for disturbed eating attitudes and behavior. This instrument has acceptable criterion-related validity, with Cronbach’s alpha ranging from 0.82 to 0.89. The internal consistency of the French version was comparable to that of the original version. The total score of the EAT-26 was considered for both the HC group and the AN group. For the HC group, the EAT-26 total score was used as a screening tool for ED, with a cut-off of 20 as recommended by the literature. For the AN group, the EAT-26 total score was considered to assess the severity of ED. The EAT was chosen due to these properties and its short duration.

History of ED and past treatments for ED. Variables regarding the history of ED (age at onset and disease duration) and past treatment for ED (history of inpatient treatment and nasogastric renutrition) were collected only for the AN group.

#### Pharmacological treatments

All participants were asked about their current use of contraception methods and psychotropic treatment.

#### Attention deficit and hyperactivity disorder (ADHD)

Two self-report questionnaires were used to screen for ADHD. The Wender-Utah Rating Scale-Child (WURS-C) was used to perform a retrospective assessment of ADHD in childhood [24, 25]. A threshold of 46/100 was defined to identify ADHD in childhood. The total score reliability is high for the WURS-2, and Cronbach’s alpha showed good reliability in the French female population (0.92) [24]. For adult participants, the WURS-C questionnaire was supplemented by the Adult ADHD Self-Report Scale (ASRS-v1.1), which screens for ADHD in adulthood with good sensitivity (68.7%), specificity (99.5%), total classification accuracy (97.9%) and kappa value (0.76) [26]. Participants younger than 18 years of age with a WURS-C score above or equal to the threshold were considered to be suffering from ADHD. Participants 18 years of age or older had to have a positive screening on the ASRS-v1.1 to be considered to be suffering from ADHD.

#### Depressive and anxiety symptoms

The Beck Depression Inventory II (BDI-II) is a self-assessment questionnaire based on 21 items that indicates the severity of depressive symptoms [27]. For each item, four response options are available, and participants were asked to choose the statement that best describes their attitudes towards the item. Scores can vary from 0 (no symptoms of depression) to 63 (maximum severity of depression). A recent meta-analysis confirmed the good psychometric properties of the BDI-II with internal consistency around 0.9 and retest reliability ranged from 0.73 to 0.96 [28].

The State-Trait Anxiety Inventory (STAI) is a self-assessment questionnaire that provides a severity of anxiety symptoms [29]. Two scores corresponding to two subscales are provided, each one made up of 20 items that measure state anxiety and trait anxiety. For state anxiety, score answers are provided on a 4-point Likert scale ranging from “no” to “yes”. For the trait anxiety scale, score answers are provided on a 4-point Likert scale ranging from “almost never” to “almost always”. The higher the score is, the worse anxiety is. A validated French version of this scale was used for our study [30]. Analyses of reliability and construct validity showed that the psychometric qualities of the French version were similar to those of the original (*α* = 0.90 for state anxiety and *α* = 0.91 for trait anxiety).

#### Impulsivity

Facets of impulsivity were assessed using the short UPPS Impulsive Behavior Scale [31]. The UPPS is a 45-item self-questionnaire that aims to measure four distinct pathways to impulsive behavior: urgency (“tendency to engage in impulsive behaviors under conditions of negative affect”), (lack of) premeditation (“difficulty in thinking and reflecting on the consequences of an act before engaging in that act”), (lack of) perseverance (“individual’s inability to remain focused on a task that may be boring or difficult”) and sensation seeking (“tendency to enjoy and pursue activities that are exciting and openness to trying new experiences that may or may not be dangerous”). The French adaptation of the UPPS, which was used in the study, has shown strong psychometric properties with Cronbach’s alpha ranging from 0.77 to 0.83 [32].

#### Cognitive evaluation

No French-specific cognitive evaluation battery was available for patients suffering from AN. For this reason, our cognitive evaluation was based on the recommendations of the National Institute of Mental Health’s Measurement and Treatment Research to Improve Cognition in Schizophrenia (MATRICS) consensus group. This battery was initially developed for schizophrenia and related disorders. More recently, and more generally, this battery has been used in different psychiatric populations, especially in ED patients [17, 33]. This battery is appropriate for comprehensively assessing basic cognitive functions to characterize the extensive cognitive domains of AN-R patients.

Intelligence quotient. The National Adult Reading Test (NART) [34] is a word-reading test widely used in clinical and nonclinical populations to estimate the level of intelligence [35]. The NART has shown good psychometric properties in measuring general intelligence and has already been used to assess premorbid ability in AN [36]. The French version of the NART (f-NART) was validated in 2005 with a good reliability in French population (*α* = 0.89) [37]. The participant had to read out a shortlist of 40 words with irregular pronunciation. The intelligence quotient can be estimated on the basis of the number of words read correctly. An equation can be used to estimate the score on the Weschler Adult Intelligence Scale-Revised (WAIS-R) [38] using the score of the f-NART. The average score (standard deviation) on the WAIS-R is 100 (15).

Central coherence. The Rey Osterrieth Complex Figure (ROCF) test [39] is a task commonly used in ED to assess central coherence [40]. The drawing strategy adopted by the participant is used to measure central coherence in two conditions (copy and delayed recall). For the copy condition, the total score, time and type of copy were recorded. For the delayed recall condition, the total score and time were recorded.

Cognitive inhibition ability. The Hayling Sentence Completion Task (HSCT) [41] is a measure of response initiation and response suppression. In the first section, the participant was required to complete as fast as possible 10 sentences (missing the last word) with the correct word. In the second section, the participant was required to complete as fast as possible 10 sentences (missing the last word) with a word unrelated to the meaning of the sentence in every way. The 30 selected sentences were validated in French [42]. The HSCT is used internationally for studies of inhibition capabilities in psychiatric conditions, such as neurodegenerative disorders. The first part evaluates the speed of response and access to the semantic stock. The second part evaluates verbal inhibition capabilities. The scores take into account the elapsed time (in seconds) for both events. For the second part, the error score is also raised. This score can vary between 0 and 45.

Cognitive flexibility. A verbal fluency task [43] was considered with phonetic verbal fluency (specific letter) and categorical verbal fluency (animal naming). The total number of correct words in 1 min for each letter and for the animal category was collected. The verbal fluency task was validated and standardized.

Planning impairment. The Tower of London (TOL) [44] was designed to assess planning impairment. In this task, two pictures (A and B) are shown to the participant. Each picture represents three colored balls (red, blue and green) positioned on three stakes of different heights. The position of the three balls differs from picture A to picture B. Participants were required to provide an answer for the minimum number of steps required to move balls from one position (picture A) to another (picture B). Each participant underwent 20 trials, and if the 20 trials were correct, 2 more difficult trials were added. For each participant, we determined the number of corrected trials over 20 (or over 22).

Episodic verbal memory. The Rey 15-item Test [45] was used to assess memory function. Rey’s 15-word test, standardized from the age of 6, corresponds to learning a list of 15 words in 5 essays. A list of 15 words is read to the participant, and she is asked to immediately recall as many words as possible. Five attempts with the immediate recall are performed. We collected the total score (total number of words found for the 5 attempts). The total score varies between 0 and 75.

Working memory. Ability in working memory is closely related to attentional abilities and plays a role in executive functions. Working memory was evaluated by an inverted span task. The backward digit span tests are used in all neuropsychological evaluation batteries, especially in the Wechsler battery [46]. The subscore is the total number of series of digits correctly repeated and varies between 0 and 28. Participants are asked to repeat a series of digits of increasing length forwards and backwards.

Attentional ability—Token Test [18, 47]. This test has the advantage of not requiring hardware. It shows results comparable to the Continuous Performance Test (comparison with 150 control participants and 150 schizophrenic patients). The subject must simultaneously put in a container two tokens (one in each hand). The subscore is based on the number of chips correctly placed in the container (that is, the number of chips in the container that were not inserted simultaneously are removed) in 60 s. The score can, therefore, vary from 0 to 100.

Speed of information processing—coding [46]. There are several validated versions, and all are taken from the Wechsler 94 code test. The subject must match, as quickly as possible, symbols to numbers from the example given. The subscore is the number of correctly assigned digits in 90 s. The version we had used, taken from the Wechsler code, was compared to symbol coding and block design, both from the Adult Intelligence Scale, 3rd edition [46]. The scale was normed, and scores varied between 0 and 110.

### Statistical analysis

Descriptive statistical analysis was conducted for both groups. Continuous variables are described by the mean and standard deviation, while categorical variables are presented as numbers and percentages. Bivariate analyses were conducted to compare the characteristics of the AN group and the HC group [48]. Logistic regression adjusted for age group was used to analyse the categorical variables, and linear regression adjusted for age group was used for the quantitative variables. A multivariate logistic regression model adjusted for age group was constructed to assess the significant differences between the AN group and the HC group using variables with *p* < 0.20 in bivariate analyses and variables with *p* > 0.20 that could influence cognitive evaluation (intelligence quotient and ADHD diagnosis). The correlation between the selected variables was tested and the variables correlated with others were removed from the multivariate model.

The analysis of homogeneity was performed with the AN group using Ward’s method [49] hierarchical ascendant classification to identify relevant subpopulations of patients with different cognitive profiles. Variables included in the hierarchical ascendant classification were age, BMI, age at onset of ED and cognitive evaluation results. Afterwards, bivariate analyses were conducted between the subpopulations of the AN group and between each subpopulation of the AN and HC groups; these analyses were performed with the Chi^2^ test for categorical variables and Kruskal–Wallis test and Dunn’s post hoc test for continuous variables.

All statistical tests were performed bilaterally with an alpha level of 5%, and *p*-values less than 0.05 were considered significant. All statistical analyses were carried out using R statistical software, version 3.1.2.

## Results

A total of 122 participants were included; these participants had a mean age of 21.0 years (SD 5.7).

### Comparison of the AN group and the HC group (Tables 1 and 2)

The bivariate analysis (Table 1) showed that patients from the AN group had significantly lower BMI and higher scores on the EAT-26 than did participants from the HC group, as expected. Psychotropic treatment was significantly more frequent for the AN group, consistent with more psychiatric morbidities (mood and anxiety disorders). Regarding cognitive evaluation, participants from the AN group and HC group did not differ in premorbid IQ, but the AN group had poorer performance on several cognitive tasks. No significant difference was found on the ROCF test (type of copy, copy score, copy time, recall score) except for the recall time. Patients performed the recall faster than the control group did (*p* = 0.02).

**Table 1:** Comparison of the AN group and HC group (*n* = 118)

	AN group (*n* = 59) *n* (%) or *m* (sd)	HC group (*n* = 59) *n* (%) or *m* (sd)	Estimate or OR	*p*-value
Age	20.7 (5.4)	21.3 (5.7)	− 0.58^a^	0.58
Eating disorders				
Current ED	59 (100)	0 (0)		–
BMI	15.2 (0.9)	21.1 (3.0)	− 5.9^a^	< 0.0001
EAT-26	32.0 (16.0)	3.9 (3.4)	2.1^a^	< 0.0001
Age at onset of ED	17.2 (5.0)	–		
Disease duration (years)	3.5 (2.9)	–		
History of inpatient treatment for ED	54 (91.5)	–		
History of nasogastric renutrition	33 (55.9)	–		
Pharmacological treatment				
Contraception method	21 (35.6)	41 (69.5)	4.8^b^	< 0.001
Psychotropic treatment	40 (67.8)	1 (1.7)	0.0^b^	< 0.0001
Psychiatric morbidity and addictive behavior				
Current mood disorders	21 (35.6)	0 (0)		–
Current suicide risk	26 (44.1)	4 (6.8)	11.1^b^	< 0.0001
Current anxiety disorders	34 (57.6)	4 (6.8)	19.4^b^	< 0.0001
Current addictive behavior	6 (10.2)	8 (13.6)	0.71^b^	0.57
ADHD	4 (6.8)	4 (6.8)	1.00^b^	1.00
Psychopathology				
BDI-II	24.6 (11.0)	6.6 (5.2)	18.0^a^	< 0.0001
STAI State	52.6 (13.5)	34.0 (10.0)	18.1^a^	< 0.0001
STAI Trait	59.7 (10.2)	39.0 (10.2)	20.7^a^	< 0.0001
Urgency (UPPS)	30.3 (7.0)	28.1 (7.0)	2.2^a^	0.11
Lack of premeditation (UPPS)	18.9 (4.5)	22.8 (4.8)	− 3.8^a^	< 0.0001
Lack of perseverance (UPPS)	17.2 (5.3)	19.4 (4.5)	− 2.3^a^	0.02
Sensation seeking (UPPS)	30.7 (7.4)	34.5 (7.5)	− 3.8^a^	0.006
Cognitive evaluation				
Premorbid intelligence quotient (WAIS score)	104.8 (7.0)	104.8 (6.3)	0.1^a^	0.94
HSCT				
Control part: time (s)	42.6 (4.0)	39.8 (3.9)	2.8^a^	0,0005
Inhibition part: score	5.6 (3.0)	4.9 (2.5)	0.7^a^	0.19
Inhibition part: time (s)	105.9 (25.5)	117.4 (29.1)	− 11.6^a^	0.03
Cognitive flexibility	54.9 (11.6)	51.3 (9.2)	3.6^a^	0.06
Planning (Tower of London)	17.1 (2.1)	18.1 (2.5)	− 1.0^a^	0.02
Episodic verbal memory	50.2 (8.3)	53.2 (7.1)	− 3.0^a^	0.04
Working memory	19.8 (3.7)	18.9 (4.0)	0.86^a^	0.24
Attentional ability	77.3 (10.6)	81.7 (10.8)	− 4.4^a^	0.04
Information processing speed	63.5 (9.3)	66.8 (10.4)	− 3.2^a^	0.07

%, percentage; *m*, mean; sd, standard deviation; ED, eating disorders; BMI, body mass index; EAT-26, Eating Attitude Test; ADHD, Attention Deficit and Hyperactivity Disorder; BDI-II, Beck Depression Inventory-II; HSCT, Hayling Sentence Completion Task; STAI, State-Trait Anxiety Inventory; UPPS, Impulsive behavior scale

^a^ Estimate, of AN group vs HC group, from multivariate linear regression adjusted on age group

^b^ Odd ratio, of AN group vs HC group, from multivariate logistic regression adjusted on age group

**Table 2:** Multivariate condition logistic regression model for comparison of the AN group and HC group (*N* = 118)

	OR	CI	*p* value
Contraception method	0.25	[0.08; 0.73]	0.01
ROCF test (recall time)	0.99	[0.98; 0.99]	0.04
Cognitive flexibility	1.08	[1.02; 1.14]	0.005
Planning (Tower of London)	0.74	[0.58; 0.93]	0.01
Information processing speed	0.93	[0.87; 0.99]	0.02
Urgency (UPPS)	1.14	[1.05; 1.24]	0.002
Lack of premeditation (UPPS)	0.75	[0.66; 0.86]	< 0.0001

*OR* odds ratio, *CI* confidence interval, *ROCF test* Rey Osterrieth Complex Figure test, *UPPS* impulsive behavior scale

Multivariate logistic conditional regression was performed (Table 2). In the first step, correlations between variables of the model were assessed, and variables correlated with STAI-state were excluded (BMI, current suicide risk, current mood disorders, current anxiety disorders, BDI, and STAI-trait). In a second step, variables that induced nonconvergence of the model were excluded (ADHD, premorbid IQ, psychotropic treatment and STAI-state). In the final multivariate logistic conditional regression model, patients from the AN group exhibited significantly better cognitive flexibility than those in the HC group when adjustments were made for other cognitive functions and impulsivity. The AN group performed significantly more poorly than the HC group did on two cognitive tasks (planning and information processing speed) when we controlled for other cognitive functions and impulsivity. The impulsivity profiles of the AN group were also significantly different from those of the HC group, with higher scores on the urgency dimension and lower scores on the lack of premeditation dimension.

### AN subpopulations (Table 3)

The hierarchical ascendant classification showed three subpopulations of AN-R: class 1 with 41 patients, class 2 with 17 patients and another class with only one patient. The results of hierarchical ascendant classification are available in Supplementary material (Figure S1). In the second step, bivariate comparisons were performed. The class with only one patient was not selected for the bivariate comparison and neither was the age-matched HC participant. Bivariate comparison of the two remaining subpopulations (class 1 cognitive impairment profile and class 2 psychopathological symptoms profile) with the HC group was performed (Table 3).

**Table 3:** Comparison of AN subpopulations (class 1 *N* = 41/class 2 *N* = 17) and HC group (*N* = 58)

	HC group (*n* = 58) *n* (%) or *m* (sd) a	AN group class 1 (*n* = 41) *n* (%) or *m* (sd) Cognitive impairment b	AN group class 2 (*n* = 17) *n* (%) or *m* (sd) Psychopathological symptoms c	*p*-value
Age	20.5 (4.9)	20.6 (4.6)	19.4 (2.7)	NS
Eating disorders				
Age at onset of ED	–	16.6 (3.7)	16.9 (2.1)	NS
Disease duration (years)	–	4.0 (3.1)	2.5 (2.1)	NS
BMI	21.1 (3.1)	15.1 (0.8)	15.3 (1.0)	AN < HC
EAT-26	3.8 (3.4)	27.2 (14.7)	44.5 (12.5)	a < b < c
Pharmacological treatment				
Psychotropic treatment	1 (1.7)	11 (26.8)	8 (47.1)	AN > HC
Psychopathology				
Urgency (UPPS)	28.3 (6.8)	29.7 (7.2)	31.3 (6.7)	NS
Lack of premeditation (UPPS)	22.8 (4.8)	19.3 (4.5)	17.2 (3.3)	AN < HC
Lack of perseverance (UPPS)	19.5 (5.0)	17.6 (5.3)	15.4 (4.5)	AN < HC
Sensation seeking (UPPS)	34.4 (7.5)	31.1 (7.6)	30.5 (6.5)	AN < HC
BDI-II	6.7 (5.1)	22.1 (11.0)	29.4 (8.5)	a < b < c
STAI state	34.7 (11.0)	47.9 (13.7)	62.2 (9.3)	a < b < c
Cognitive evaluation				
Premorbid intelligence quotient (WAIS score)	104.6 (6.2)	102.6 (6.0)	109.9 (5.6)	a & b < c
Cognitive flexibility	51.3 (9.3)	53.0 (10.8)	61.3 (9.8)	a & b < c
Attentional ability	81.5 (10.8)	75.9 (11.2)	81.9 (6.8)	b < a & c
Planning (Tower of London)	18.2 (2.5)	16.9 (2.2)	17.9 (1.5)	b < a & c
Information processing speed	67.0 (10.3)	60.5 (7.4)	71.8 (7.7)	b < a & c
ROCF test (recall time in sec)	128.8 (46.4)	97.8 (44.3)	131.8 (35.2)	b < a & c
Working memory	18.9 (4.0)	19.7 (3.5)	20.6 (3.4)	NS
Episodic verbal memory	53.3 (7.1)	49.4 (8.6)	52.2 (7.9)	NS
HSCT (error score)	5.0 (2.5)	6.0 (3.0)	4.5 (2.9)	NS

%, percentage; *m*, mean; sd, standard deviation; ED, eating disorders; BMI, body mass index; EAT-26, Eating Attitude Test; BDI-II, Beck Depression Inventory-II; HSCT, Hayling Sentence Completion Task; ROCF test, Rey Osterrieth Complex Figure test; STAI, State-Trait Anxiety Inventory; UPPS, Impulsive behavior scale; NS, no significant difference neither within AN subpopulations nor between AN subpopulations and the HC group; AN < HC, significant difference between both AN subpopulations and the HC group, with HC scores significantly higher than AN scores and no significant difference between AN subpopulations; AN > HC, significant difference between both AN subpopulations and the HC group, with HC scores significantly lower than AN scores and no significant difference between AN subpopulations; a < b < c, all groups are significantly different from one to another, with HC scores significantly lower than AN class 1 scores and AN class 1 scores significantly lower than AN class 2 scores; a & b < c, no significant difference between HC scores and AN class 1 scores, but AN class 2 scores are significantly higher than HC scores and AN class 1 scores; b < a & c, no significant difference between HC scores and AN class 2 scores, but AN class 1 scores are significantly lower than HC scores and AN class 2 scores

#### Comparison between AN subpopulations (Table 3)

The two AN subpopulations were similar for current age, age at onset of ED, disease duration, current BMI, psychotropic treatment and impulsivity (UPPS scores), but they were different for anxio-depressive symptoms and cognitive performance. Indeed, the hierarchical ascendant classification highlighted a small proportion of patients (class 2 with 17 patients—psychopathological symptoms profile) with higher ED severity, anxiety and depression symptoms than the other patients (class 1 with 41 patients—cognitive impairment profile). Regarding cognitive evaluation, class 2 patients (psychopathological symptoms profile) had better scores than class 1 patients (cognitive impairment profile) did for premorbid IQ, cognitive flexibility, attentional ability, planning impairment and information processing speed. Central coherence was similar for both classes of patients except for recall time: class 1 patients spent less time achieving the ROCF than class 2 patients did. No significant differences between AN subpopulations were highlighted for working memory, episodic verbal memory and cognitive inhibition ability.

##### Comparison between AN class 1 patients (cognitive impairment profile) and HC participants (Table 3)

As expected, AN class 1 patients had lower BMI and higher scores on ED scale severity than HC participants did. AN class 1 patients also exhibited more anxious and depressive symptoms and less impulsivity than HC participants did. Regarding cognitive evaluation, premorbid IQ and cognitive flexibility were similar between AN class 1 patients and HC participants, but AN class 1 patients performed more poorly than HC participants did on attentional ability, planning impairment and information processing speed. Central coherence was similar except for recall time, where class 1 patients spent less time achieving the ROCF than HC participants did. No significant differences between AN class 1 patients and HC participants were reported with the other cognitive tasks.

##### Comparison between AN class 2 patients (psychopathological symptoms profile) and HC participants (Table 3)

AN class 2 patients had lower BMI and higher scores on ED scale severity, anxious symptoms and depressive symptoms than HC participants did. Regarding cognitive evaluation, premorbid IQ and cognitive flexibility were significantly better for AN class 2 patients than for HC participants. No significant differences between AN class 2 patients and HC participants were reported with other cognitive tasks.

## Discussion

First, the study shows the differences between control and AN-R patients in several cognitive functions. This result confirms our hypothesis that AN-R patients have cognitive impairment in information processing speed and planning. Second, this study highlights two subgroups of patients, one with more marked cognitive impairment and a second with more psychopathological symptoms.

For the first point, the results of this study confirm our hypothesis that AN-R patients have cognitive impairments in information processing speed and planning, consistent with recent data from the literature [50, 51] when we compared their performance to a healthy control group of the same age. Contrary to our expectations, we did not find any difference in premorbid intelligence quotient (IQ) or central coherence. More surprisingly, the patients performed better on mental flexibility.

In our sample, the premorbid intelligence quotient (IQ) of the patients was comparable to that of the control group. The question of the IQ of patients with AN is still debated, with the measures varying according to the populations studied and the scales used (current evaluation of the intelligence quotient or premorbid evaluation) [36]. It seems that the premorbid IQ is more relevant to assess because of the intricacies between active cognitive impairment of the disease and the IQ assessment itself.

This study did not find an impairment in central coherence, based on the Rey Osterrieth Complex Figure test, except for recall time. Patients recalled faster than the controls did. Central coherence is a complex cognitive capacity to integrate information into a global meaning. This ability is based on preserved executive functions and preserved social cognition abilities. Our result and the contradictory data of the literature could be explained by the lack of a specific evaluation tool [52, 53]. On the other hand, the improved capacity for recall could be explained by their higher score for urgency on the UPPS.

In our sample, patients exhibited significantly better cognitive flexibility than the control group did when adjustments were made for other cognitive functions and impulsivity. This result is surprising but can be explained by the age of the group and the dimensional perfectionism of AN-R patients [54]. Indeed, young age does not allow us to prejudge the final level of education, which is correlated with the result of mental flexibility [55]. To better explore these data, we would have to match the level of education of the parents, which the design of our exploratory study did not allow.

The strength of this study is the identification of two subgroups of patients. The first subgroup had more marked cognitive impairment (in attentional abilities, speed of information processing, planning, verbal memory and mental flexibility) and fewer psychopathological symptoms than did the second subgroup of the patients and the control group. For some researchers, this cognitive impairment could be mainly due to a state of hypervigilance that saturates the working memory of information and prevents patients from filtering information and adapting to environmental stimuli [14].

The second subgroup of patients, on the other hand, had more psychopathological symptoms in the anxiety and depression domains and fewer cognitive impairments than the first subgroup of patients and the control group did. These data showing high levels of mood and anxious comorbidity are congruent with recent data in the literature [56, 57]. Moreover, the second subgroup had an even greater performance on mental flexibility than the control group did. These two subgroups were comparable on the impulsivity profile evaluated with the UPPS: their scores on the search for novelty, lack of premeditation and lack of perseverance were lower than the respective scores of the control group. They had a less impulsive profile than the control group did but a sense of urgency comparable to that of the control group.

This is the first time, to our knowledge, that two distinct profiles have been identified in patients suffering from AN-R. We hypothesize that the two subgroups have two different expressions of the same pathology. The first subgroup of patients would have a clinical presentation more related to a state of hypervigilance and hyperarousal, while the second group of patients would have a clinical presentation more related to a state of hyper control and perfectionism [58]. This hypothesis reinforces the notion that anorexia nervosa could be associated with both the autistic clinical spectrum and the obsessional clinical spectrum [59, 60].

Finally, this hypothesis would make it possible to explain the contradictory results of studies on the effectiveness of current cognitive remediation programs [61, 62]. Cognitive remediation programmes and emotion-centred cognitive therapies show potential for improving the progressive prognosis of patients suffering from AN-R [63, 64]. In light of our data, it would seem appropriate to propose two types of specific cognitive remediation programmes to the patients concerned (i.e., a program more focused on cold cognitions and a distinct second program more focused on emotions and hot cognitions) rather than only one cognitive remediation programme (which may not be relevant depending on the patient’s profile).

However, this study suffers from several limitations. Although a fatigability bias cannot be completely excluded, it is important to note that the duration of the assessment was between 1 and 1 h and a half, which is shorter that of a complete neuropsychological assessment in a clinical sample (including major cognitive disorder). Second, our recruitment was limited to a specific geographical area, which does not allow us to extend our results to all patients with AN-R. In addition, our recruited population mainly included hospitalized patients, i.e., those with a more severe profile of the disease. The size of our sample was modest, especially for the analysis into the two subgroups of patients (with a possible lack of power). For the comparison of AN subgroups, the use of tools such as the Eating Disorder Inventory (EDI-3) or Eating Disorder Examination Questionnaire (EDE-Q) might be of interest to assess other dimensions associated with ED, such as perfectionism. Finally, this study did not specifically evaluate social cognition or metacognitive processes. These cognitive functions seem to be interesting to evaluate with regard to our analysis in subgroups. These limits, however, were counterbalanced by the rigorous recruitment of our sample (inclusion and exclusion criteria, matching for age) and the choice of valid cognitive tests used in the clinical population and recommended by the MATRICS Consensus Group. Concerning the Ravello Profile, which is a neuropsychological test battery for anorexia nervosa [65], no validated French version was available at the time our study was conducted. Moreover, the cognitive domains explored in our study correspond to those recommended in the Ravello Profile (IQ, executive functioning inhibition, fluency, switching, planning, flexibility, central coherence and visual perception).

## Conclusion

Although this study does not find any impairment of central coherence in patients with AN-R, our results highlight other specific cognitive impairments in these patients with normal premorbid IQ. Two distinct patient profiles appear to emerge: one profile with a more pronounced cognitive impairment on cold cognitions and a second profile with more psychopathological symptoms of anxiety and depression. These results warrant confirmation by larger studies with a more specific evaluation of the impact of emotional status [66, 67]. In clinical practice, these results open up perspectives for the development of more specific cognitive remediation programs (one specific program for cold cognitions and another specific program targeting emotions and hot cognitions).

### What is already known on this subject

Several studies have examined the cognitive profiles of patients as a potential facilitating and maintenance factor of eating disorders. But specific data in restrictive AN remain contradictory.

### What this study adds

Two distinct patient cognitive profiles emerge which could explain the contradictory previous results of literature and open up perspectives for the development of more specific cognitive remediation programs.

## Electronic supplementary material

Below is the link to the electronic supplementary material.

Supplementary material 1 (DOC 136 kb)

## Data Availability

All participants provided written informed consent (for those under 18 years old, written informed consent was provided by a legal representative) for using data for this study with data processing and storage in the secure database from Nantes University Hospital. They did not provide non opposition for the use of data for furthers researches. However, we are willing to make our data available upon request as we consider that it is important for open and reproducible science, and thus we will ensure that all interested and qualified researchers will be able to be granted access.
