# Supplementary material for: Evaluation of cognitive impairment in a French sample of patients with restrictive anorexia nervosa: two distinct profiles emerged with differences in impaired functions and psychopathological symptoms
Source: Eat Weight Disord. 2020 Aug 7;26(5):1559–70. doi: 10.1007/s40519-020-00981-w (PMC8128741; doi:10.1007/s40519-020-00981-w)
Supplement: Supplementary file 1 — Supplementary material 1 (DOC 136 kb) [file 40519_2020_981_MOESM1_ESM.doc]

**Fig. S1. Hierarchical ascendant classification of AN group (N= 59)**

*
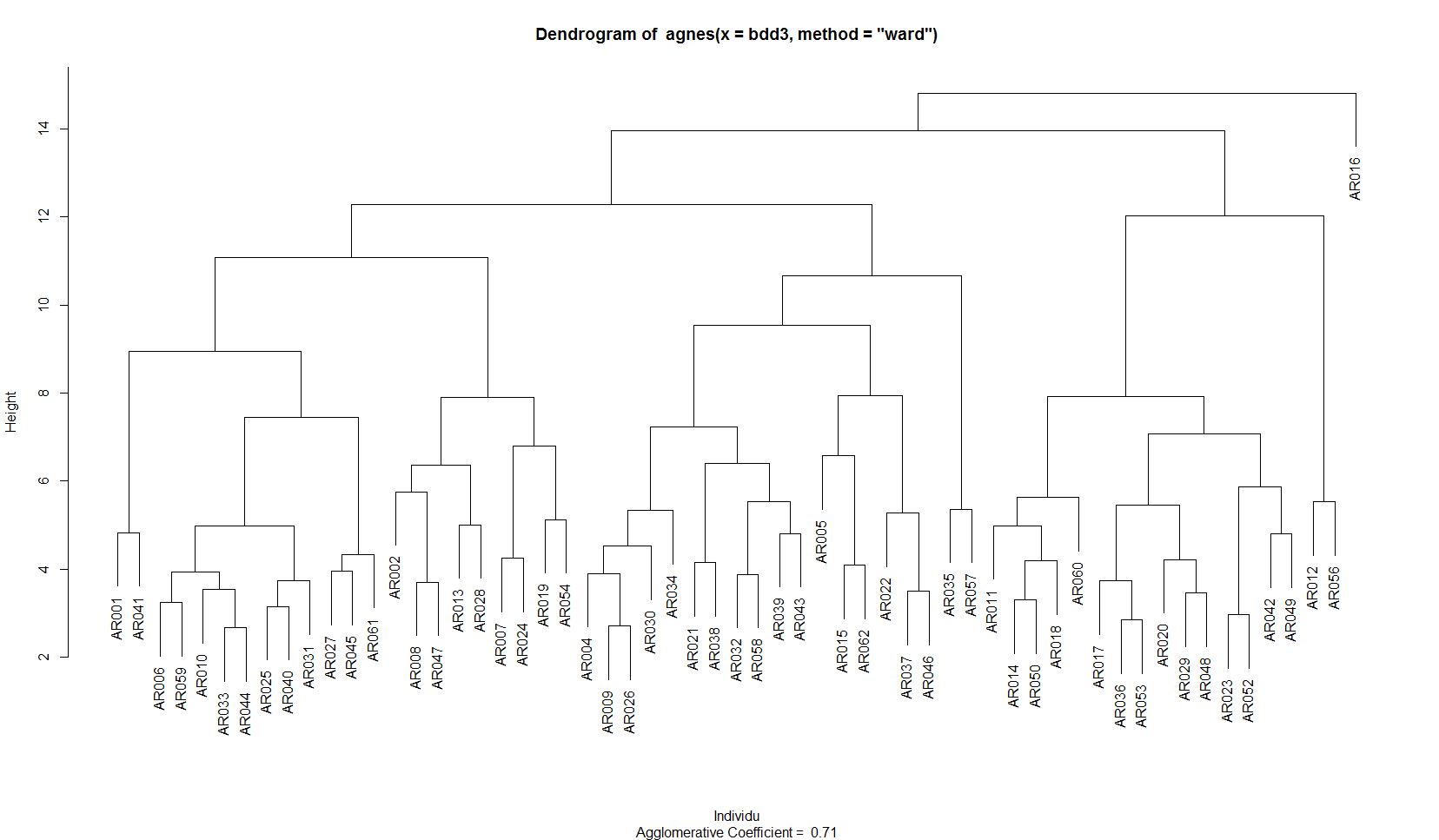
*

AN class 1 subpopulation n=41

Cognitive impairment profile

AN class 2 subpopulation n=17

Psychological symptom profile

AN, anorexia nervosa.
